# Tongue Cyst in a Neonate: Unusual Presentation

**DOI:** 10.7759/cureus.9252

**Published:** 2020-07-18

**Authors:** Ayshah J Al-mahboob, Eman A Hajr, Ahmed Alammar

**Affiliations:** 1 Otolaryngology - Head and Neck Surgery, King Abdul-Aziz University Hospital, Riyadh, SAU; 2 Department of Ear, Nose and Throat, Imam Mohammad Ibn Saud Islamic University, Riyadh, SAU; 3 Otolaryngology - Head and Neck Surgery, King Saud University Medical City, Riyadh, SAU

**Keywords:** tongue, cyst, airway, obstruction, stridor, vallecular

## Abstract

Congenital cysts of the tongue base are an uncommon cause of airway obstruction. The diagnosis of upper airway cysts requires a high index of clinical suspicion. We report a case of a vallecular cyst that uniquely extended to the dorsum of the tongue, and the patient presented with airway distress. We found that this presentation may facilitate an early diagnosis, as asymmetry of the tongue can be picked up easily during proper clinical examination.Therefore, inspection and palpation of the dorsal surface of the tongue is crucial for the approach of pediatric patients with airway obstruction.

## Introduction

Congenital cysts of the tongue base are a fairly uncommon cause of airway obstruction [1,2]. Patients with vallecular cysts can present with feeding difficulties, failure to thrive, stridor, and cyanotic episodes [2]. Seriously but infrequently, patients may present with sudden upper airway obstruction, which can result in death [2].

Therefore, the diagnosis of upper airway cysts requires a high index of clinical suspicion because these cysts have a high potential for morbidity and mortality [3].

Anatomically, laryngeal cysts are divided into true vallecular cysts and vallecular pseudocysts depending on their location - whether they are close to the lingual surface of the epiglottis or at the base of the tongue, respectively [4]. The interesting aspect of our case is that the vallecular cyst extended to the dorsal surface of the tongue, which to our knowledge, has not been reported previously in the literature. We present an uncommon case of a vallecular cyst with translingual extension presenting with respiratory distress (treated surgically) and discuss the diagnosis and management of the same.

## Case presentation

A 40-day-old baby girl (40 weeks gestational age) was delivered by cesarean section after an uncomplicated pregnancy and delivery. Her birth weight was 3 kg, and she was discharged from the nursery along with the mother four days after delivery in good health. She was doing fine until the age of one month when her mother noticed a swelling on the left side of her tongue that progressed in size and resulted in breathing difficulties, mainly during sleep. Positioning the baby on the left lateral side improved breathing. She also had difficulties with cyanotic spells during feeding. After seeking medical advice, the patient was diagnosed with an oral mass with compression to the airway and was then referred to our hospital for further management. The patient was intubated and received nasogastric tube (NGT) feeding.

**Figure 1:**
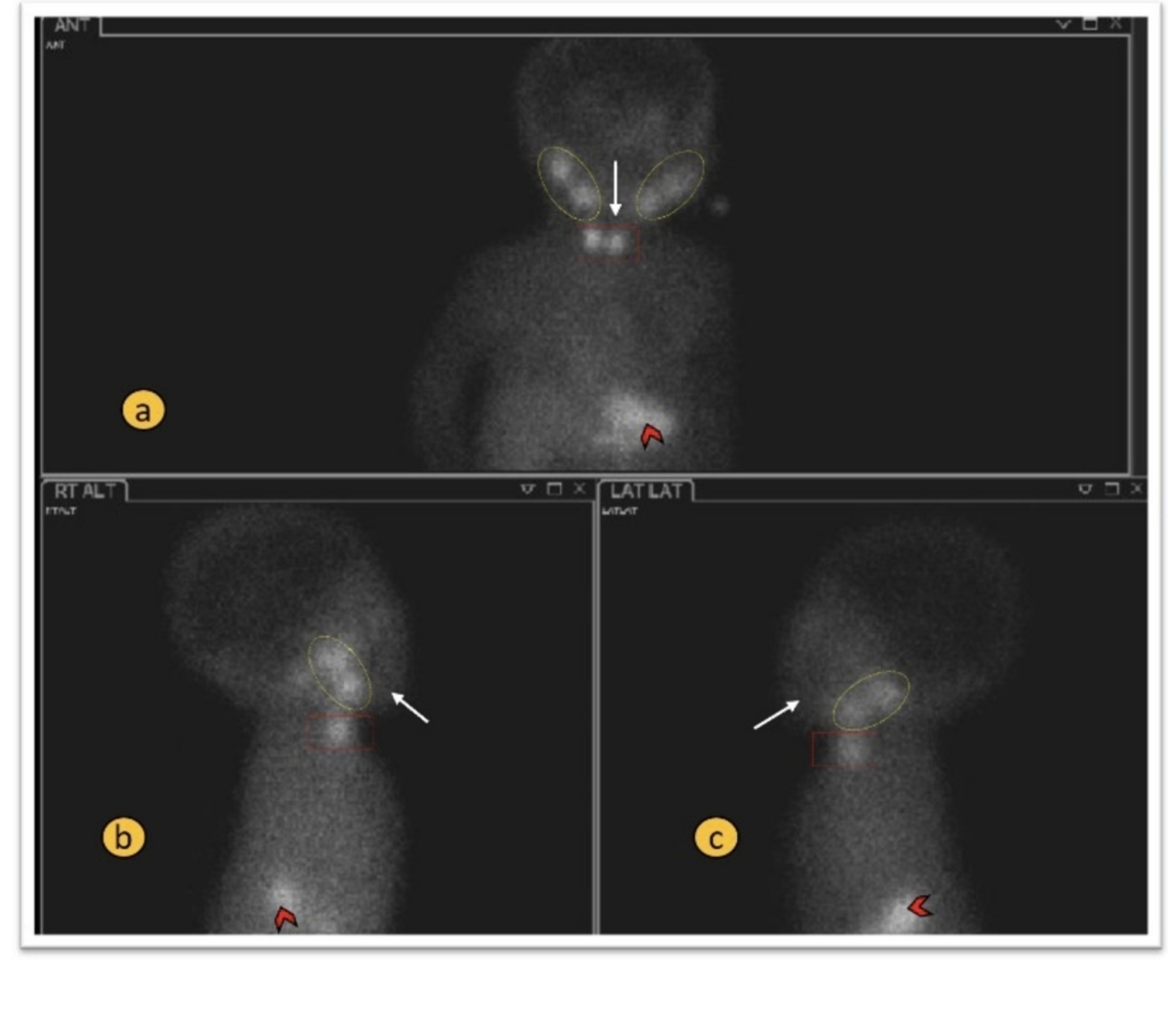
Normal radionuclide thyroid scan - anterior and lateral views Panels a-c: Showing physiological radionuclide uptake in the salivary glands bilaterally (dotted ovals) and stomach (arrowheads).The thyroid gland is in the normal location and showing normal homogenous uptake (red rectangle). There is no radio tracer uptake in the region of chin or base of tongue (white arrows).

**Figure 2:**
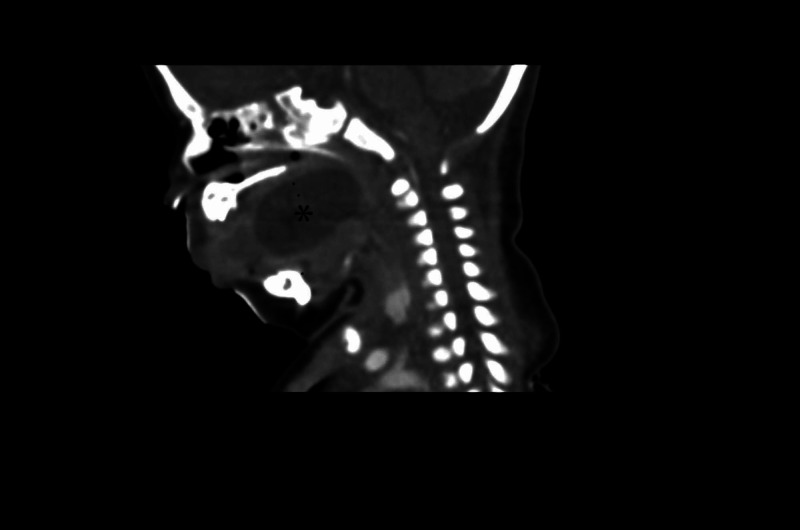
Sagittal CT scan of the neck with contrast demonstrating the hypodense cystic lesion at the posterior aspect of oropharynx (asterisk)

**Figure 3:**
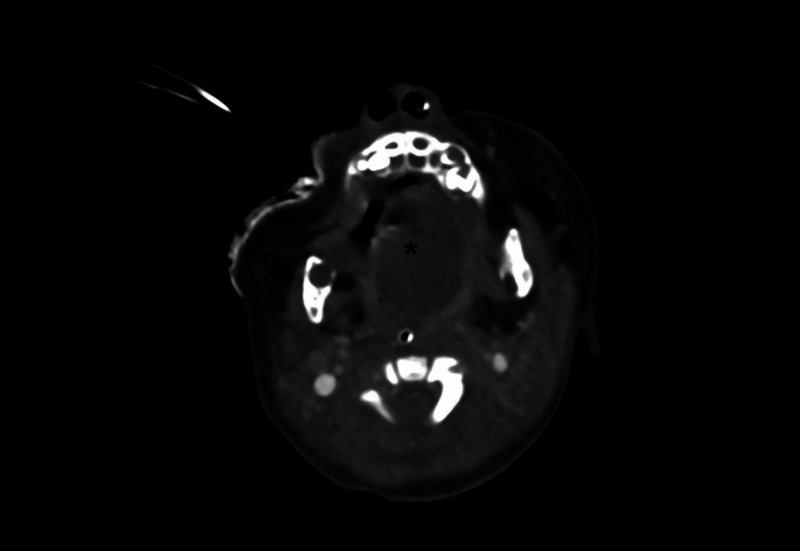
Axial CT scan of the neck with contrast showing mild peripheral rim enhancement measuring almost 3.4 x 2.5 x 2.2 cm (asterisk)

Regarding investigations, routine blood tests were normal, including thyroid function tests. The thyroid scan was normal (Figure 1). Computerized tomography (CT) scans are shown in Figures 2-3.

Her clinical picture was consistent with a large oral cyst extending from the base of the tongue to the left side of the dorsum. The risks and benefits of surgical intervention were fully explained to her parent, and after obtaining surgical consent, she was taken to the operating room for surgical excision.

**Figure 4:**
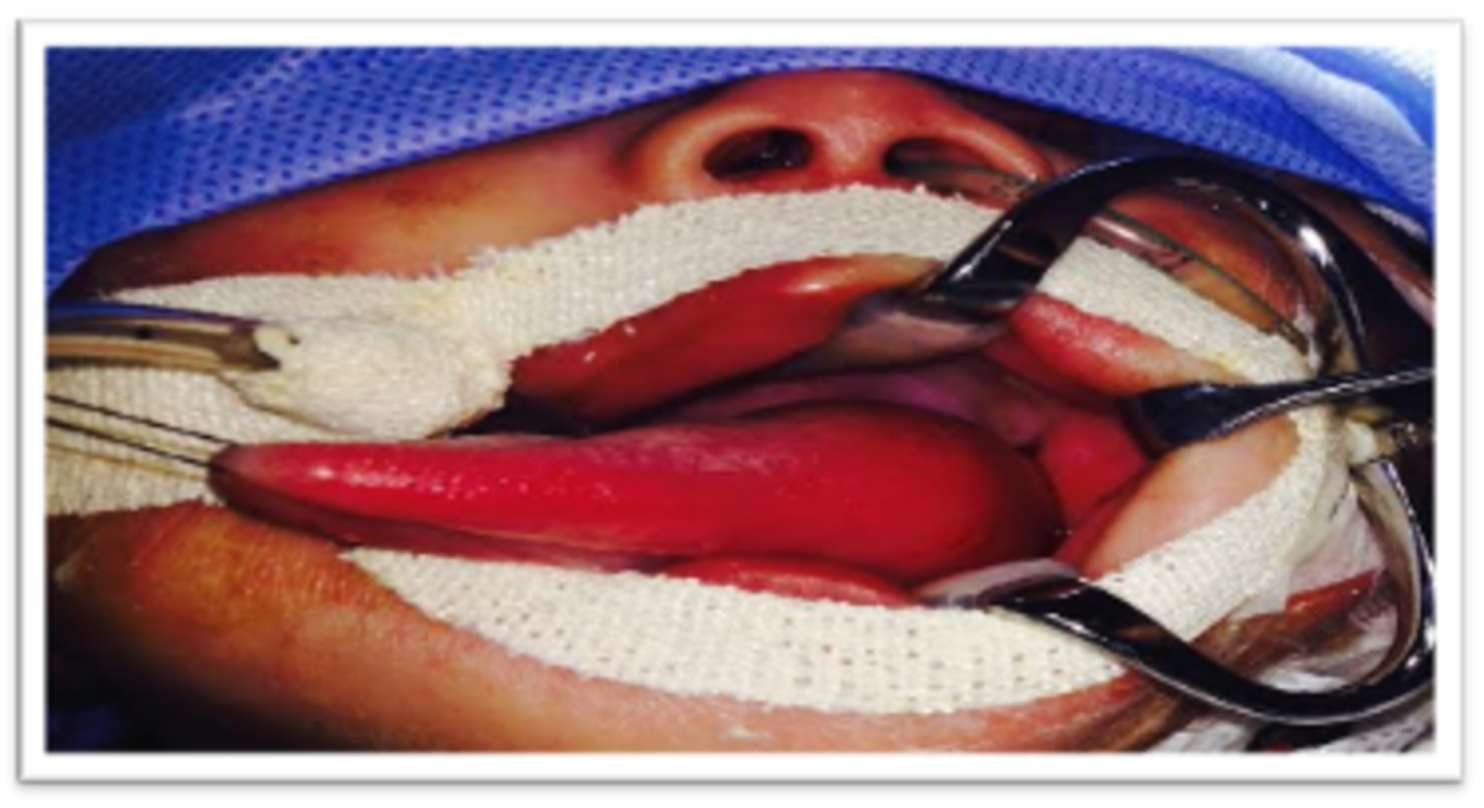
Intraoperative photo showing a swelling extending from the base of the tongue to the left side of the dorsum

The patient was in the supine position and was intubated with a 3.5 mm endotracheal tube (ETT). The mouth was opened using a self-retaining retractor. The tongue was pulled out utilizing a suture at the tip. The cystic mass was visualized and palpitated, measuring approximately 3 x 3 cm on the left side of the tongue, extending to the tongue base (Figure 4). Aspiration of the content revealed approximately 4 ml of clear fluid, which was sent for cytology; the result was consistent with a mucus retention cyst and was negative for malignant cells. A 1.5 cm incision was made using a mono-polar Colorado needle (Stryker Inc., Kalamazoo, MI) on the left lateral aspect of the tongue at the roof of the cyst after injecting 1 ml of 1% lidocaine in 1:100000 epinephrine. Part of the superior wall of the cyst was excised and sent for histopathology, which showed the content of the cyst: macrophages, degenerate epithelial cells, and debris.

The patient remained intubated for one day and was then extubated smoothly. She was kept for observation for one week without any respiratory problems or feeding difficulties and was discharged home in good condition.

One month later, when she was seen in the clinic, she had recurrence of the collection that was smaller in size. Therefore, the patient consented for excision of the tongue cyst and was intubated under general anesthesia smoothly with a 3.5 mm ETT. The mouth was opened with a self-retaining retractor and the tongue was retracted utilizing a 2.0 silk suture at the tip. A 2 x 4 cm cystic mass was palpated at the junction of the posterior 2/3 and anterior 1/3 of the left lateral side of the tongue. Then, marsupialization was performed through a 3 cm incision at the midline of the cyst using a mono-polar Colorado needle, and the edges were sutured utilizing an inverted (Vicryl) suture in such a way that the cyst remained open to drain freely. The patient was extubated smoothly and was observed in the postoperative period to be doing fine with no complications.

At the two year follow-up, the patient had no recurrence and was doing fine.

## Discussion

Congenital cysts of the tongue base are an uncommon cause of airway obstruction. Their incidence in Eastern countries is almost 1.82 per 100,000 live births per year [1]. Cysts of the tongue base are an important differential diagnosis in neonates and infants who present with stridor, respiratory distress, and failure to thrive [5]. In the literature, multiple cases of supra-glottic cysts have been reported in the pediatric age group, and vallecular cysts represent the identities that are most likely close to our case, as can be noticed from our patient's imaging that demonstrated a trans-lingual cystic lesion extending to the tongue base. 

Strider, as a well-known symptom in pediatric patients, has multiple causative conditions. Laryngomalacia represents the most common type. Interestingly, the presence of a supraglottic mass can coexist with laryngomalacia when the mass effect compresses the adjacent epiglottic cartilage and causes supraglottic prolapse [6].

Airway management can be troublesome, especially for patients with a vallecular cyst [7]. As illustrated in our case, the airway was the priority, and it was secured before the referral for definitive management.

The characteristics of vallecular cysts that determine the mode of presentation are the position and the size, mostly the size, and patients with smaller cysts are usually asymptomatic [2]. In our case, the probable cause of airway obstruction, in addition to the size, was the extension of the cyst to the dorsal surface of the tongue (Figure 4). 

Flexible laryngoscopy or bronchoscopy are considered the diagnostic tools for vallecular cysts. Therefore, special attention should be paid to the valleculae, as they can be easily missed and lead to misdiagnosis [8].

Early diagnosis of vallecular cysts remains a challenge. Some authors report that large numbers of vallecular cysts are diagnosed at autopsy [9]. Holinger found that four months is usually the average length of time until the correct diagnosis is made by endoscopy [10].

However, we expect patients with a vallecular cyst who have a similar presentation as our case to have an early diagnosis since the tongue swelling is easily noted by the parents, as was done in our case. Therefore, early medical advice and management can be obtained. Additionally, asymmetry of the dorsal tongue can be clearly picked up during proper clinical examination, even for inexperienced physicians.

Surgical decompression is the treatment of choice. Cyst marsupialization is usually adequate and results in a satisfactory outcome. Simple aspiration or drainage with a simple incision, however, has a high chance of recurrence, as observed in our case [4].

## Conclusions

Vallecular cyst is considered a rare and unusual cause of stridor and respiratory distress in children. Early diagnosis remains a challenge and it requires a high index of clinical suspicion. We report a case of a vallecular cyst that uniquely extended to the dorsum of the tongue, and the patient presented with airway distress. Therefore, inspection and palpation of the dorsal surface of the tongue is crucial for approaching pediatric patients with airway obstruction; it can help physicians narrow their differential diagnosis and hence can help with early diagnosis and intervention.
